# The evaluation of cardiac functions in deep Trendelenburg position during robotic-assisted laparoscopic prostatectomy

**DOI:** 10.3389/fmed.2023.1273180

**Published:** 2023-09-25

**Authors:** Emir Kılınç, Serap Aktas Yildirim, Halim Ulugöl, Elif Eroğlu Büyüköner, Bülent Güçyetmez, Fevzi Toraman

**Affiliations:** ^1^Department of Anesthesiology and Reanimation, Faculty of Medicine, Acibadem University, Istanbul, Türkiye; ^2^Department of Cardiology, Faculty of Medicine, Acibadem University, Istanbul, Türkiye

**Keywords:** general anesthesia, cardiac efficiency, arterial elastance, ventriculo-arterial coupling, hemodynamic monitoring

## Abstract

**Objective:**

This study aimed to demonstrate the reliability of the cardiac cycle efficiency value through its correlation with longitudinal strain by observing the effect of the deep Trendelenburg position.

**Design:**

A prospective, observational study.

**Setting:**

Single center.

**Participants:**

Between May and September 2022, the hemodynamic parameters of 30 patients who underwent robotic assisted laparoscopic prostatectomy under general anesthesia were prospectively evaluated.

**Measurements and main results:**

All invasive cardiac monitoring parameters and longitudinal strain achieved transesophageal echocardiography were recorded in pre-deep Trendelenburg position (T3) and 10th minute of deep Trendelenburg position (T4). Delta values were calculated for the cardiac cycle efficiency and longitudinal strain (values at T4 minus values at T3). The estimated power was calculated as 0.99 in accordance with the cardiac cycle efficiency values at T3 and T4 (effect size: 0.85 standard deviations of the mean difference: 0.22, alpha: 0.05). At T4, heart rate, pulse pressure variation, cardiac cycle efficiency, dP/dt and longitudinal strain were significantly lower than those at T3 (*p* = 0.009, *p* < 0.001, *p* < 0.001, and *p* < 0.001, respectively). There was a positive correlation between the delta-cardiac cycle efficiency and delta-longitudinal strain (*R*^2^ = 0.36, *p* < 0.001).

**Conclusion:**

Although the absence of significant changes in mean arterial pressure and cardiac index after Trendelenburg position suggests that cardiac workload has not changed, changes in cardiac cycle efficiency and longitudinal strain indicate increased cardiac workload due to increased ventriculo-arterial coupling.

## Introduction

Robotic Assisted Laparoscopic Prostatectomy (RALP) is a surgical procedure that provides very good short-term results and is considered the gold standard in prostate cancer surgery because it is minimally invasive (1, 2). The pneumoperitoneum and deep Trendelenburg position (dTD) (at least 25°–45° upside down) required for RALP surgeries can cause significant pathophysiological changes in both the pulmonary and cardiac systems.

Although the Trendelenburg position is a life-saving maneuver in hypovolemic patients, it also carries unwanted risks. Cardiac output (CO) can be maintained by increasing venous return in the dTD. However, an increase in intrathoracic pressure due to the applied intraperitoneal pressure may cause impaired venous return and a decrease in CO (3). In addition, the changing heart configuration in the dTD may cause an increase in the workload of the heart (4, 5). Therefore, we need to evaluate hemodynamic management with more advanced monitoring techniques, including fluid therapy in the perioperative period in patients undergoing RALP (6).

Cardiac workload may increase and efficiency may decrease in cases where the pressure volume relationship changes from physiological to pathological. Therefore, ventriculo-arterial coupling (VAC) is a more accurate approach for evaluating the cardiac and vascular system together. VAC is expressed as the ratio of arterial elastance (Ea) to ventricular elastance (Ees) (VAC:Ea/Ees) (7). VAC is a physiological term that characterizes an interaction between the left ventricular contractility and the afterload posed by the arterial system. Another parameter that provides information about the efficiency and performance of the heart and calculated from arterial wave analysis is Cardiac Cycle Efficiency (CCE). CCE is an index that calculates the efficiency of the heart over energy consumption (energy expended in systole/total energy: W(t)sys/W(t)beat × K(t)). Refers to the cardiovascular system’s ability to maintain homeostasis at different energy levels (8). Many studies have highlighted the importance of CCE in monitoring cardiac performance (9–11). However, we still have limited data on how cardiac functions change in the dTD.

In this study, we aimed to evaluate the effect of the dTD applied in RALP surgeries on cardiac functions, with the CCE and longitudinal strain (LS) [Transesophageal Echocardiography (TEE) parameter], and to show the correlation between CCE and LS.

## Materials and methods

### Patients

After obtaining approval from the Acıbadem Mehmet Ali Aydınlar University Local Ethics Committee (Decision No; 2022-20/05) and signing an informed consent document, 30 patients older than 18 years of age who were scheduled for RALP were included in the study. Patients younger than 18 years old, with heart failure, valvular disease, rhythm disorders, a history of myocardial infarction (MI) in the last 3 months, and those who did not agree to give their consent were excluded from the study.

### Study protocol

Before the induction of general anesthesia, radial artery cannulation was performed in all patients, and invasive arterial pressure monitoring was performed with the MostCare(^®^) (Vytech, Vygon, Padua, Italy) device.

After induction of anesthesia with 2 mg/kg IV propofol and 1 μg/kg IV remifentanil bolus, all patients were given 0.6 mg/kg IV rocuronium for neuromuscular blockade and intubated after mask ventilation. Following orotracheal intubation, the respirotary rate was adjusted such that the tidal volume was 8 ml/kg and the end-expiratory carbon dioxide (EtCO2) was 35 mmHg. Positive end-expiratory pressure (PEEP) was set to 5 cmH2O and mechanical ventilation was started in volume-controlled (VCV) mode.

Anesthesia was maintained by inhalation of an oxygen/air mixture with 40% end-expiratory oxygen percentage (EtO2) and sevoflurane inhalation with a minimum alveolar concentration (MAC) of 0.9–1. The continuity of muscle relaxation was provided with 0.1 mg/kg/h IV rocuronium infusion, and the continuity of analgesia was provided with 0.5 μg/kg/min IV remifentanil infusion. The depth of anesthesia was monitored using a bispectral index (BIS monitor; Covidien Medical, Boulder, CO, USA) and a range of 40–60 was targeted.

The TEE probe was placed in all patients after anesthesia induction and intubation. Echocardiographic data were digitally collected, recorded and analyzed using a software analysis program (EchoPAC v. 112, GE Healthcare Vingmed Ultrasound AS, Horten, Norway). Echocardiographic imaging and analysis were performed by an experienced cardiologist. In accordance with the guidelines of the American Society of Echocardiography (ASE), LS was calculated by averaging the values measured at the segmental level within the same frame (12). Analysis for LS included the evaluation of three echocardiographic images from standard mid-esophageal 4-chamber, commissural and long-axis images (0, 60, and 120°). Before anesthesia induction (T1) and after anesthesia induction (T2), after pneumoperitoneum was applied in the supine position (T3), in the dTD at the 10th minute (T4), in the dTD at the 1st hour (T5), after returning to the supine position (T6) and extubation after (T7) pulse wave analysis parameters and demographic data of the patients were recorded; TEE measurements were performed at T3 and T4.

### Statistical analysis

The sample size of the prospective observational study was calculated based on the change of the CCE values before and after dTD. Accordingly, it was determined that 30 patients should be included in the study in order for the mean difference of the CCE values at both times to be 0.15, standard deviations: 0.20 and α = 0.05 and power: 0.80.

Descriptive parameters are presented as means ± standard deviations, medians (quartiles), and percentages. Shapiro–Wilcox test was used to test the normal distribution. Paired-student t, Wilcoxon rank and Freidman tests were used to compare measured parameters before and after dTD position; the Pearson correlation test was used to determine the correlation between CCE and LS. Statistical analyses were performed using SPSS version 28.0. A *p*-value less than 0.05 was accepted for statistical significance.

## Results

The median age of patients included in the study was 64. While 80% of the patients had an ASA score of ≥ 2, 63.3% had a history of hypertension (Table 1). Postoperative troponin levels were within the normal range and no cardiac complications were observed.

**TABLE 1:** Patient characteristics.

Patients, *n*	30
Age, years	64 ± 7
BMI, (kg/m^2^)	27.4 ± 3.6
ASA ≥ 2, *n* (%)	24 (80.0)
**Comorbidities, *n* (%)**	
Hypertension	19 (63.3)
Diabetes Mellitus	9 (30.0)
Coronary arterial disease	5 (16.7)
COPD	3 (10.0)
**Preoperative period,**	
Fasting (h)	12 (10–14)
Hemoglobine, (g dL^–1^)	14.0 ± 1.5
Hematocrit, (%)	41.7 ± 4.2
Urea (mg dL^–1^)	31 ± 12
Creatinine (mg dl^–1^)	1.1 (1.0–1.1)
eGFR, (ml min^–1^ 1.73 m^–2^)	79 ± 13
**Intraoperative drug use,**	
Ephedrine (total dose), (mg) Noradrenaline, (mcg kg^–1^ min^–1^)	10 (5–25)
**Intraoperative adverse events, *n* (%)**	
Cardiac event,	0 (0.0)
Arrhythmia,	0 (0.0)
Hypotension,	2 (6.7)
ST depression,	0 (0.0)
**Postoperative Troponin levels,(ng ml^–1^)**	
at 24th h	0.004 (0.003–0.007)
at 48th h	0.006 (0.004–0.010

ASA, American society of anesthesiology; BMI, body mass index; COPD, chronic obstructive pulmonary disease; eGFR, estimated glomerular filtration rate.

The LS values measured by TEE at the 10th minute (T4) of the patients’ dTD were found to be significantly lower than those before the Trendelenburg position (T3) (*p* < 0.001) (Table 2).

**TABLE 2:** Comparisons of hemodynamic parameters between T_3_ and T_4_.

	T_3_ (Before dTB)	T_4_ (10 Min. After Dtb)	*p*
Heart Rate, (/dk)	55 (50–64)	54 (49–57)	0.009
SAP, (mmHg)	114 ± 24	116 ± 15	0.659
MAP, (mmHg)	81 ± 16	85 ± 11	0.277
PPV, (%)	10 (7–13)	7 (4–9)	0.002
Ea (mmHg ml^–1^)	0.95 (0.74–1.18)	1.01 (0.88–1.09)	0.621
CI (L/min/m^2^)	2.4 ± 0.4	2.4 ± 0.3	0.751
SVI (ml/m^2^)	43.6 ± 12.3	44.9 ± 9.1	0.428
CPO (W)	0.89 ± 0.25	0.92 ± 0.21	0.710
CCE (unit)	0.15 ± 0.21	−0.05 ± 0.24	<0.001
dP/dt_*max*_ (mmHg s^–1^)	0.72 ± 0.21	0.58 ± 0.17	<0.001
**TEE parameters**			
LS (%),	−17.3 ± 2.6	−14.4 ± 3.2	<0.001

CCE, cardiac cycle efficiency; CI, cardiac indeks; CPO, cardiac power output; Ea, arterial elastance; MAP, mean arterial pressure; PPV, pulse pressure variation; SAP, systolic arterial pressure; SVI, stroke volum index; LS, longitudinal strain.

At the 10th minute (T4) of the dTD, heart rate, Pulse Pressure Variation (PPV), CCE and dP/dt were significantly decreased compared to T3 (Table 3).

**TABLE 3:** Comparison of intraoperative parameters according to time points.

	T_1_ (Before AI)	T_2_ (After AI)	T_3_ (Before dTB)	T_4_ (10 min. after dTB)	T_5_ (1 hr. After dTB)	T_6_ (Before Extubation)	T_7_ (After Extubation)	*P*
Heart Rate/dak	67 (58–75)	63 (56–76)	55 (50–64)	54 (49–57)**	55 (50–63)	64 (48–71)	69 (59–77)	<0.001
SAP (mmHg)	163 (14–171)	106 (94–127)^ßßß^	119 (92–137)	114 (102–129)	98 (84–111)^##^	98 (75–114)	129 (122–147)^ΩΩΩ^	<0.001
MAP (mmHg)	106 (100–111)	74 (69–89)^ßßß^	81 (69–98)^$^	85 (76–92)	74 (63–82)^#^	69 (56–80)	90 (83–94)^ΩΩΩ^	<0.001
Ea (mmHg ml^–1^)	1.00 (0.82–1.16)	1.02 (0.87–1.16)	0.95 (0.74–1.18)	1.01 (0.88–1.09)	0.96 (0.69–1.16)	0.93 (0.70–1.10)	0.97 (0.86–1.25)	0.902
PPV (%)	9 (5–13)	11 (8–12)	10 (7–13)	7 (4–9)**	14 (7–15)^##^	8 (7–12)	7 (3–16)	0.012
CI (L/min/m)	3.4 (2.8–4.0)	2.2 (2.1–2.6)^ßßß^	2.5 (2.2–2.8)	2.4 (2.2–2.6)	2.3 (2.1–2.6)	2.1 (1.9–2.3)	2.8 (2.5–3.3)^ΩΩ^	<0.001
CCE (unit)	0.32 ± 0.41	0.08 ± 0.23	0.15 ± 0.21	−0.05 ± 0.24***	−0.11 ± 0.37	−0.03 ± 0.43	0.14 ± 0.27	<0.001
dP/dt_*max*_ (mmHg s^–1^)	1.6 (1.3–1.8)	0.8 (0.7–1.0)^ßßß^	0.8 (0.6–0.9)	0.6 (0.5–0.7)***	0.5 (0.4–0.6)^##^	0.5 (0.4–0.7)	1.0 (0.8–1.1)^ΩΩΩ^	<0.001

AI, anesthesia induction; CCE, cardiac cycle efficiency; CI, cardiac index; dTB, deep-Trendelenburg; Ea, arterial elastance; MAP, mean arterial pressure; PPV, pulse pressure variation; SAP, systolic arterial pressure. Friedman test was used for comparison of all groups. Paired student-*t* and Wilcoxon rank tests were used for pairwise comparisons of each group with the previous group. In pairwise comparisons, *p*; 0.05–0.01 for 1 symbol; *p*; 0.01–0.001 for 2 symbols; and *p* < 0.001 for 3 symbols were used.

**FIGURE 1:**
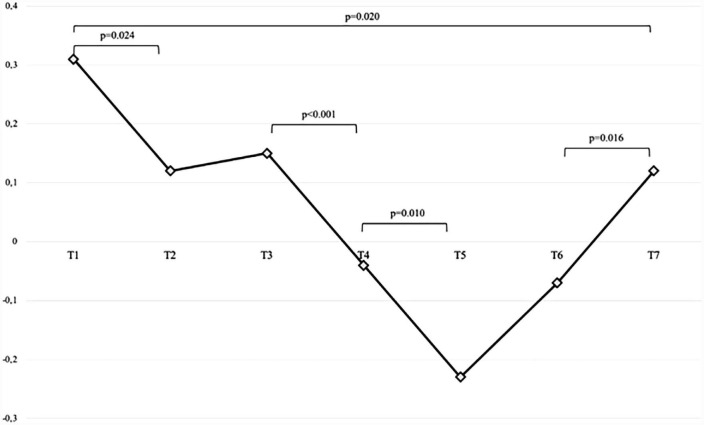
CCE change in the intraoperative period. T1, before induction anesthesia; T2, after induction anesthesia; T3, after pneumoperitonenum is a administered in the supine position; T4, 10th minute after deep trendelenburg position; T5, 1st hour after deep trendelenburg position; T6, after returning to the supine position; T7, post extubation.

The CCE values of the patients decreased with the induction of anesthesia (T2) and reached their lowest level at the 10th minute of the Trendelenburg position (T4) (*p* < 0.001) (Figure 1).

**FIGURE 2:**
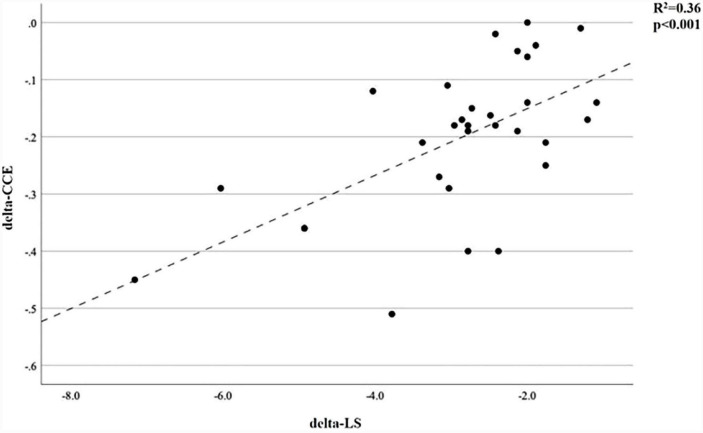
Correlation between delta CCE and delta LS. CCE, cardiac cycle efficiency; LS, longitudinal strain. Delta values = T4 values-T3 values.

It has been shown that there is a significant positive correlation between the delta values calculated from the difference of the CCE and LS values measured at the 10th minute before and after Trendelenburg (*R*^2^ = 0.36 *p* < 0.001) (Figure 2).

## Discussion

To our knowledge, this is the first study to evaluate hemodynamic stability by using CCE in patients undergoing RALP.

“In major surgery patients, it becomes difficult to have information about the volume status by monitoring the blood pressure.” Therefore new monitoring parameters have entered our clinical practice by defining the interaction between the heart and vascular system and showing the efficiency of the work done by the heart. One of these, CCE, is a parameter that defines the interaction of the heart and arterial system in terms of energy consumption and efficiency. When the coupling between the ventricle and arterial system is disrupted, the cardiac efficiency also decreases. Therefore, CCE is an important indicator of the VAC (13).

Longitudinal strain indicates the percentage of dimensional deformation that occurs in the heart in the longitudinal axis, is a preload-independent indicator of cardiac contractility and is associated with cardiac efficiency (14, 15). Similar to CCE, LS is an important indicator of VAC (16–18).

In this study, we aimed to determine the energy consumption and efficiency of the heart in the Trendelenburg position by monitoring these two parameters.

Ventriculo-arterial coupling is a parameter calculated using the Ea/Ees formula, and it provides the opportunity to evaluate both the cardiac and vascular systems together, as it shows the transfer of the energy formed in the Left Ventricle (LV) to the vascular system. When the VAC is impaired, the optimal cardiovascular combination that enables the maximum mechanical energy transfer from the ventricle to the arterial system will also be impaired, which will reduce the working efficiency of the heart (19, 20). Many researchers have analytically shown that the efficiency is optimal when Ea/Ees is close to 1 (21, 22). In physiological conditions if Ea/Ees > 0.5, it is expressed as a situation where cardiac efficiency decreases and energy consumption increases and therefore VAC is impaired.

The VAC considers the complex relationship between ventricular and arterial system elastances and exhibits a strong correlation with cardiac work and cardiac efficiency (23–26). Studies have shown a negative correlation between LV mechanical efficiency and VAC (Ea/Ees) (13). VAC impairment is an early indicator of systolic dysfunction. Therefore, CCE, an important indicator of VAC, provides much more accessible bedside information as well as echocardiography in terms of showing cardiovascular status (27).

In our study, LS (−17.3 ± 2.6, −14.4 ± 3.2, < 0.001) and CCE (0.15 ± 0.21, −0.05 ± 0.24, < 0.001) values decreased with the transition from the supine position (T3) to Trendelenburg position (T4) after pneumoperitoneum was applied. We found that the blood pressure and Cardiac Index (CI) values did not change. These values show us that the heart spends more energy to maintain the same pressure and volume values.

In our study, PPV changes in T3 and T4 [10 (7–13), 7 (4–9), 0.002] show that venous return increased in the Trendelenburg position but stroke volume (SV) did not change, indicating that LV end diastolic volume (EDV) and end systolic volume (ESV) increased. Meanwhile, the blood pressure values did not change, suggesting that the end systolic pressure (ESP) did not change either (ESP = Systolic Arterial Pressure (SAP) × 0.9). The ratio of left ventricular ESP to ESV provides Ees, an indicator of LV contractility (28). ESP remained unchanged with Trendelenburg position, ESV increased, and therefore Ees and cardiac contractility decreased.

The dP/dt_*max*_ is another important indicator of contractility (29). The significant decrease in dP/dt_*max*_ and LS values between these two time periods (T3-T4) also supports this decrease in contractility. dP/dt_*max*_ correlates with CCE in that it represents the arterial reflection of cardiac work. There are many studies in the literature showing the correlation of CCE and dP/dt_*max*_ (10, 27). Monge Garcia et al. (29) found a significant correlation between dP/dt_*max*_ and Ees in their study and reported that it is possible to follow the changes in Ees using dP/dt_*max*_. We believe that this decrease in contractility is caused by the baroreflex mechanism that develops because of the activation of pressure-sensitive baroreceptors in the atria, carotid sinuses and aortic arch after venous return (30, 31). Despite increased venous return after Trendelenburg position, contractility decreased. This resulted in the absence of an increase in CI and Mean Arterial Pressure (MAP) consistent with the increase in preload. Thus the energy efficiency of the heart is impaired.

In our study, the LS decreased significantly between T3 and T4. Strain is a quantitative representation of the dimensional deformation created by the force in a material. It is also defined as the relative change in the basal size of objects due to stress or applied force (12, 32). It should be interpreted as a percentage and absolute value. For example, a LS value change from −20 to −15%, indicated a decrease in the amount of strain. This indicated a change in the negative direction (12).

Ruppert et al. (17) investigated the effect of pressure and volume loading on LS and VAC, and showed that both volume loading and pressure loading reduced LS and this change was related to VAC.

Longitudinal strain is an indicator of LV systolic function and studies have shown that it is correlated with VAC and therefore negatively correlated with Ees (16–18, 33). However, when the absolute value of LS is interpreted, it shows a positive correlation with Ees and a negative correlation with VAC similar to CCE. In our study, there was a volume overload in the Trendelenburg position and the LS decreased. This once again revealed that the VAC value increased and the coupling between the cardiac and vascular systems was impaired. The negative correlation between LS and VAC observed in our study is consistent with other studies (17, 18, 33, 34).

We found a positive correlation when we compared the amount of change between the two time periods of CCE and LS (Figure 2). This correlation shows that the two parameters (CCE and LS), which are negatively correlated with VAC, change in the same direction during the Trendelenburg position, and that CCE, which is less invasive than LS, can be used as an easily applicable parameter in the evaluation of VAC. The perioperative changes in the CCE values in our study were also remarkable (Figure 1). It decreased significantly between T1 and T2 because of sympathetic blockade due to anesthesia induction. Because, as shown in studies, the negative inotropic effect of propofol is greater than its effect on systemic vascular resistance (35). For the reasons explained above, it also decreased significantly between T3 and T4 and continued to decrease until the Trendelenburg position ended and returned to the supine position (T6) again. These changes show that when factors such as dTD, pneumoperiontum, fluid restriction and long surgical time are added, more than standard monitoring parameters are required for hemodynamic management in major surgery. Even when parameters such as CI, MAP and Heart Rate (HR) are within normal ranges, the cardiac workload may have increased and the heart may have become more inefficient.

To date, little is known about the effects of the dTD and pneumoperitoneum on the heart and arterial system. Although there are studies in the literature showing that cardiac functions changes, the exact mechanism of the change in the relationship between the ventricle and the arterial system is unclear (4, 36, 37). Most studies in the literature did not detect a significant change in cardiac parameters during the Trendelenburg position (3, 4, 38, 39). Our study shows that with the transition to the Trendelenburg position, the coupling between the ventricle and the arterial system is disrupted, which affects cardiac efficiency. Therefore, we believe that the VAC may be impaired due to increased workload and energy expenditure during Trendelenburg position, especially in patients with limited cardiac functions. We believe that it is important to know that these changes cannot be monitored with using standard monitoring parameters (HR and SAP). We suggest that CCE, which correlates well with LS, can be used as an indicator of VAC in clinical practice.

The limitations of this study are that cardiac risky patients were not included and the follow-up period was limited.

## Conclusion

In conclusion, in our study, which included the risks related to major surgery, dTD and pneumoperitoneum, there was no significant change in HR, SAP, MAP and CI with Trendelenburg position. These daha show us that double product of heart (HRxSAP), which is also an index of myocardial oxygen consumption, did not change significantly. Despite double product of heart suggest that cardiac workload does not change, changes in CCE and LS indicate that cardiac workload is increased due to impaired VAC. Observing of advanced monitoring parameters may be a routine procedure for hemodynamic monitoring in major surgeries using Trendelenburg position and pneumoperitoneum. There is a need for both experimental and clinical studies to be conducted in this area, with more comprehensive and larger patient numbers.

## Data availability statement

The raw data supporting the conclusions of this article will be made available by the authors, without undue reservation.

## Ethics statement

The studies involving humans were approved by the Acıbadem University and Acıbadem Healthcare Institutions Medical Research Ethics Committee (ATADEK). The studies were conducted in accordance with the local legislation and institutional requirements. The participants provided their written informed consent to participate in this study.

## Author contributions

EK: Writing–original draft, Writing–review and editing. SY: Writing–review and editing. HU: Writing–review and editing. EB: Writing–review and editing. BG: Writing–review and editing. FT: Writing–review and editing.
